# Aquaporin 9 inhibits growth and metastasis of hepatocellular carcinoma cells via Wnt/β-catenin pathway

**DOI:** 10.18632/aging.102698

**Published:** 2020-01-22

**Authors:** Shengtao Liao, Hongyu Chen, Min Liu, Li Gan, Chuanfei Li, Wenguang Zhang, Lin Lv, Zhechuan Mei

**Affiliations:** 1 Department of Gastroenterology, The Second Affiliated Hospital of Chongqing Medical University, Chongqing 400010, P.R. China; 2 Department of Gastroenterology, University-Town Hospital of Chongqing Medical University, Chongqing 401331, P.R. China; 3 Teaching and Research Section of Forensic Medicine, College of Basic Medicine, Chongqing Medical University, Chongqing 400016, P.R. China; 4 Department of Gastroenterology, Banan People’s Hospital of Chongqing, Chongqing 401320, P.R. China

**Keywords:** aquaporin 9, hepatocellular carcinoma, prognosis, Wnt/β-catenin pathway

## Abstract

Hepatocellular carcinoma (HCC) is the most common type of liver cancer worldwide, and it is the second leading cause of cancer-related mortality. Aquaporin 9 (AQP9) is an essential aquaporin in the liver and located in the basolateral membrane of hepatocytes, but its roles on HCC has not been completely elucidated. This study investigated the regulatory functions of AQP9 in the pathogenesis of HCC. The expression levels of AQP9 were significantly down-regulated in HCC tissues and cells, which was also correlated with tumor size and number, TNM stage, five-year survival rate, lymphatic and distal metastasis within the patients. Furthermore, overexpressed AQP9 suppressed the proliferation, migration and invasion of HCC cells. The levels of PCNA, E-cad, N-cad, α-SMA, DVL2, GSK-3β, cyclinD1 and β-catenin in HCC cells were reduced by overexpressed AQP9, while cell apoptosis was remarkably enhanced. Additionally, following the treatment with Wnt/β-catenin signaling inhibitor (XAV939), the proliferative activity of HCC cells was significantly inhibited; PCNA and EMT-related markers were down-regulated; migration and invasion of cells were notably suppressed; cell apoptotic rate was decreased. Vice versa, after the cells were treated with Wnt/β-catenin inducer (SKL2001), the effects caused by overexpressed AQP9 were abrogated. *In vivo* studies indicated that tumor volume and weight were remarkably decreased in AQP9 overexpression group, where the levels of Wnt/β-catenin signaling- and EMT-associated molecules were also reduced. Taken together, our results suggested that overexpressed AQP9 could inhibit growth and metastasis of HCC cells via Wnt/β-catenin pathway. AQP9 may be a promising therapeutic target for the treatment of patients with HCC.

## INTRODUCTION

The incidence of hepatocellular carcinoma (HCC) is constantly rising, and it has become the one of the leading causes of cancer-related mortality among Chinese population [1, 2]. HBV infection is still considered as the major cause of HCC globally, and 50-80% of HCC is associated with HBV infection [3]. In addition, as the obesity rate gradually increases, NAFLD may also contribute to the occurrence of HCC [4]. The mechanisms underlying the development of HCC is complex due to the heterogeneity of HCC. Only 10-13% of HCC patients can be cured by liver transplantation and surgical treatment [5]. Therefore, liver cancer poses great threat to public health globally. At present, the initiation and progression of liver cancer are still under investigation, which involve multiple genes and signaling pathways, but the underlying mechanisms are not completely elucidated.

Aquaporins (AQPs) consist of six transmembrane helices and two non-transmembrane helices [6]. Tissue- and organ-specific expression of AQPs are found in the digestion system. AQP-1, -8 and -9 are abundantly expressed in the liver, bile duct and spleen [7]. AQP9 is a type of glycoprotein with the molecular weight of ~31 kDa that has been recently discovered [8]. AQP9 was predominantly expressed in the basolateral membrane of mammalian liver cells and served essential roles on the absorption of arsenite, whose accumulation could lead to damaged liver cells and HCC [8]. Furthermore, the expression levels of AQP9 in the basolateral membrane of liver cells was reduced in extrahepatic cholestasis induced by bile duct ligation, indicating that AQP9 could participate in the transport of bile [9]. In addition, AQP9 is involved in the metabolism of glycerol in the liver; glycerol is produced by triacylglycerols catabolism in the adipose tissue, and it enters the liver via portal vein and participates in glyconeogenesis [10]. In H4IIE cells, insulin was able to inhibit the expression of AQP9 in a time- and dose-dependent manner and insulin could suppress the expression of AQP9 by binding to the -496/-502 promoter region [11]. Therefore, AQP9 is involved in numerous liver-related diseases.

Previous studies have indicated that the Wnt/β-catenin signaling pathway serves essential roles during cell differentiation, proliferation and apoptosis [12]. Wnt/β-catenin signaling is associated with various biological processes such as the regulation of epithelial cell phenotype, intercellular junctions, and tissue homeostasis. Impairment in the abovementioned pathway could contribute to EMT. E-cadherin (E-cad) is required for the formation of intercellular junctions, and depletion of E-cad could lead to tumor invasion and metastasis. Wnt family plays essential roles during cell proliferation, differentiation, adhesion and migration in β-catenin-dependent or -independent manner [13]. Disrupted Wnt signaling may contribute to the development of cancer. The classical Wnt/β-catenin pathway is characterized by the accumulation of cytoplasmic β-catenin, that interacts with TCF/LEF-Legless-PYGO DNA binding proteins to form a complex of transcriptional activators [14]. This process is involved in the transcriptional regulation of numerous oncogenes including MMP-7, Myc, Cyclin D1, CD44, Twist and Snail. However, whether AQP9 is able to function through Wnt/β-catenin pathway remains unknown.

In the present study, AQP9 was significantly down-regulated in HCC tissues and cells and associated with the prognosis of HCC patients. Further functional study revealed that overexpressed AQP9 was able to inhibit proliferation, migration and invasion of HCC cells via Wnt/β-catenin pathway, and suppressed tumor growth *in vivo*.

## RESULTS

### AQP9 was down-regulated in HCC tissues and associated with the prognosis of patients

**Figure 1:**
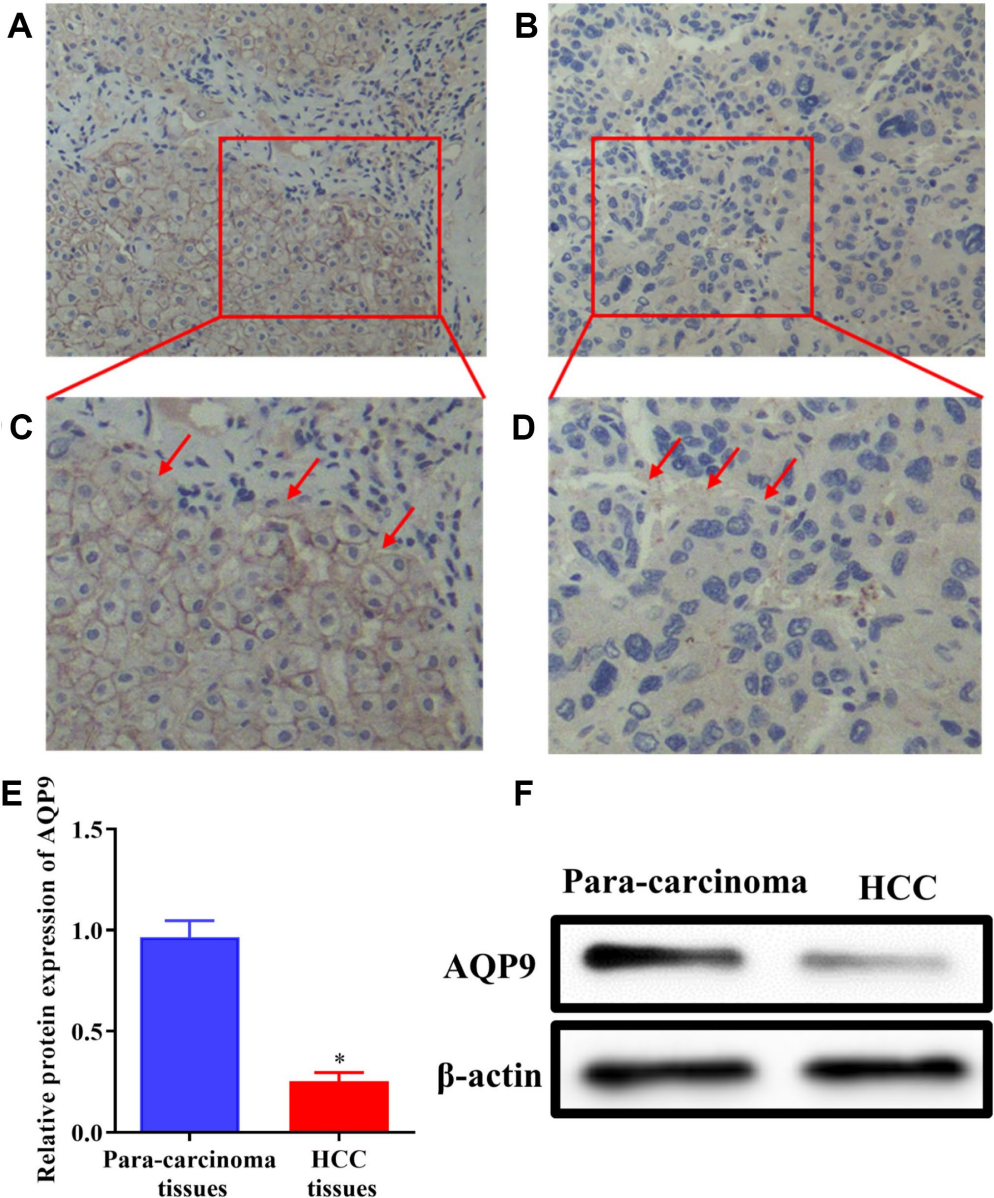
**The expression of AQP9 was reduced in HCC samples.** (**A**–**D**) The levels of AQP9 were examined in HCC and paired non-tumor tissues by immunohistochemistry analysis (magnificationx200 and x400). (**E** and **F**) The levels of AQP9 were also examined using western blotting. The results were represented as mean ± SD. P<0.05 vs. para-carcinoma control. Each experiment was repeated 3 times. AQP, aquaporin 9; HCC, hepatocellular carcinoma.

**Figure 2:**
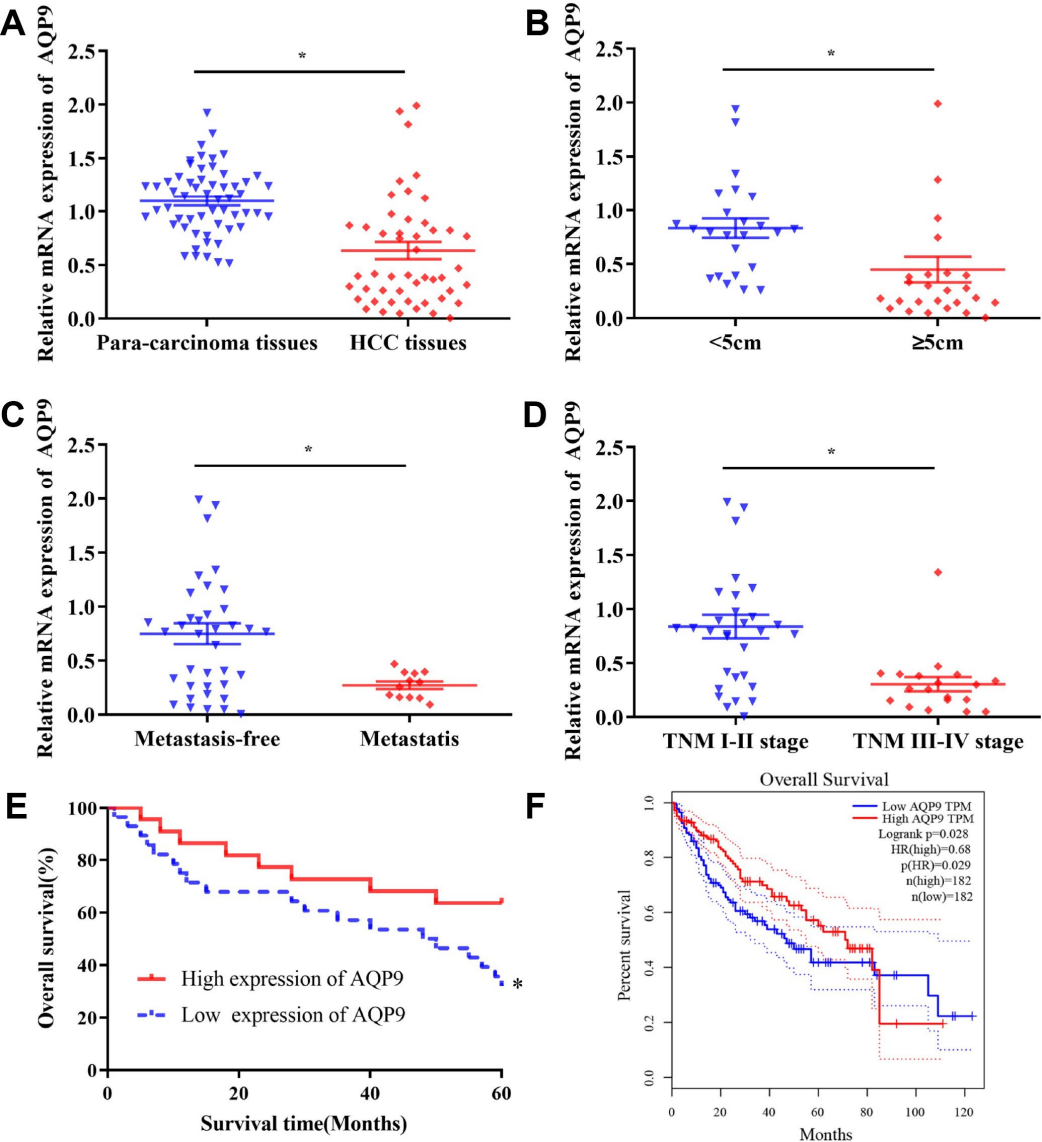
**The levels of AQP9 in HCC tissues were associated with the prognosis of patients.** (**A**) The expression of AQP9 was evaluated in HCC and matched para-carcinoma samples by RT-qPCR. The levels of AQP9 were examined in HCC tissues with various tumor diameter (**B**) lymph node metastasis (**C**) and different TNM stages (**D**). Survival rate of 50 HCC patients with different AQP9 expression levels (**E**) and those in the GEPIA database (**F**); 182 cases with high-/low-expression were analyzed. The results were represented as mean ± SD. P<0.05 vs. control group. Each experiment was repeated 3 times. AQP, aquaporin 9; HCC, hepatocellular carcinoma; RT-qPCR, reverse transcriptase-quantitative polymerase chain reaction.

AQP9 was abundantly expressed on the cell membrane in para-carcinoma tissues (Figure 1A and 1C), while its expression level was significantly decreased in HCC samples (Figure 1B and 1D). Western blotting was also used to determine the expression of AQP9 protein in HCC and matched para-carcinoma tissues. The results revealed that the expression level of AQP9 was remarkably down-regulated in HCC samples compared with the control (Figure 1E and 1F). To further investigate the relationship between AQP9 expression and the progression of HCC, RT-qPCR was performed to examine the levels of AQP9 mRNA. The results indicated that AQP9 mRNA levels were significantly reduced in HCC tissues compared with paired para-carcinoma controls (Figure 2A). Furthermore, the levels of AQP9 were evaluated in patients with different clinical characteristics. The expression of AQP9 was significantly down-regulated in HCC patients with larger tumor diameter (≥5cm), lymph node metastasis and advanced TNM stage (III-IV; Figure 2B–2D). In univariate analysis, low expression of AQP9 was associated with tumor size/number, TNM stage and lymph node/distant metastasis within HCC patients (Table 1).

**Table 1:** Relationship between AQP9 expression levels and clinical characteristics in HCC patients.

**Parameters**	**Case number (n=50)**	**AQP9 expression levels**		**χ^2^**	***P***
		**Low expression(n=28)**	**High expression(n=22)**		
Age (Year)				0.141	0.707
<50	19(38.0%)	10(35.7%)	9(40.9%)		
≥50	31(62.0%)	18(64.3%)	13(59.1%)		
Gender				0.000	0.988
Male	42(84.0%)	23(82.1%)	19(86.4%)		
Female	8(16.0%)	5(17.9%)	3(13.6%)		
Tumor size				13.487	**0.000**
<5cm	24(48.0%)	7(25.0%)	17(77.3%)		
≥5 cm	26(52.0%)	21(75.0%)	5(22.7%)		
Tumor number				10.959	**0.001**
Single	19(38.0%)	5(17.9%)	14(63.6%)		
Multiple	31(62.0%)	23(82.1%)	8(36.4%)		
Tumor grade				0.542	0.462
Poor differentiation	14(28.0%)	9(32.1%)	5(22.7%)		
Middle or high differentiation	36(72.0%)	19(77.9%)	17(77.3%)		
TNM stage				16.213	**0.000**
I-II	31(62.0%)	10(35.7%)	21(95.5%)		
III-IV	19(38.0%)	18(64.3%)	1(4.5%)		
Portal vein tumor thrombus				3.328	0.068
Without	41(82.0%)	20(71.4%)	21(95.5%)		
With	9(18.0%)	8(28.6%)	1(4.5%)		
Lymph node metastasis				12.158	**0.000**
Without	38(76.0%)	16(57.1%)	22(100.0%)		
With	12(24.0%)	12(42.9%)	0(0.0%)		
Distant metastasis				4.266	**0.039**
Without	40(80.0%)	19(67.9%)	21(95.5%)		
With	10(20.0%)	9(32.1%)	1(4.5%)		
AFP				1.172	0.279
<400μg/L	41(82.0%)	21(75.0%)	20(90.9%)		
≥400μg/L	9(18.0%)	7(25.0%)	2(9.1%)		
Cirrhosis				1.096	0.295
Without	30(80.0%)	15(53.6%)	15(68.2%)		
With	20(80.0%)	13(46.4%)	7(31.8%)		

A total of 50 HCC patients were followed up for five years. The results suggested that there was no significant difference in one-/three-year survival rates between AQP9 low- and high-expression group. However, the five-year survival rate of AQP9 low-expression group was 35.7% (10/28), which was significantly reduced compared with AQP9 high-expression group (63.6%, 14/22; Table 2). The log-rank test revealed that the overall survival rate of AQP9 low-expression group was remarkably poorer compared to high-expression counterpart (Figure 2E). To further determine the relationship between AQP9 expression and the prognosis of HCC patients, 182 patients with AQP9 high-expression and 182 patients with AQP9 low-expression were analyzed in GEPIA (Figure 2F). The data indicated that the survival rate of AQP9 low-expression group was notably decreased, suggesting the occurrence and progression of HCC was correlated with the expression of AQP9.

**Table 2:** Relationship between AQP9 expression levels and survival rate in HCC patients.

**Overall survival**	**Low expression of AQP9(n=28)**	**High expression of AQP9(n=22)**	**χ^2^**	***P***
1 Year	20(71.4%)	19(86.4%)	1.624	0.203
3 Years	16(57.1%)	16(72.7%)	1.375	0.241
5 Years	10(35.7%)	14(63.6%)	4.079	**0.043**

### AQP9 was down-regulated in HCC cells

**Figure 3:**
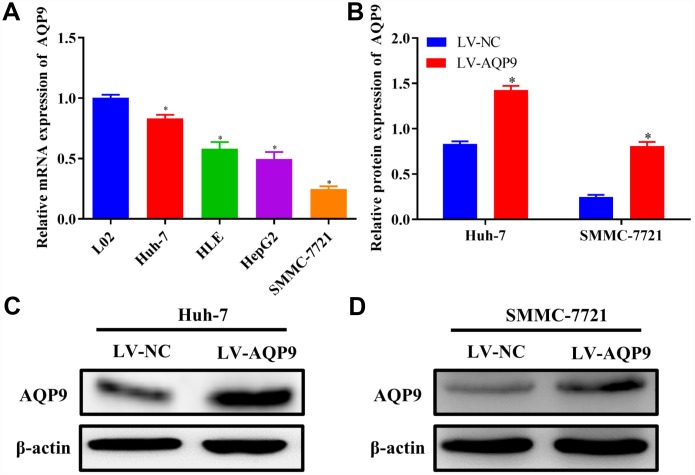
**Down-regulation of AQP9 in HCC cells.** (**A**) The mRNA levels of AQP9 in HCC cell lines were determined compared with the control. (**B**–**D**) The protein levels of AQP9 in Huh-7 and SMMC-7721 cells transfected with LV-NC or LV-AQP9 were also examined using western blotting. The results were represented as mean ± SD. P<0.05 vs. LO2 or LV-NC. Each experiment was repeated 3 times. AQP, aquaporin 9; HCC, hepatocellular carcinoma; NC, negative control.

To further evaluate the expression of AQP9 in HCC cell lines, the levels of AQP9 mRNA were examined in normal liver cells L02 and HCC cell lines Huh-7, HLE, HepG2 and SMMC-7721 by RT-qPCR. The expression level of AQP9 was significantly reduced in HCC cells compared with normal hepatocytes L02 (Figure 3A). Additionally, Huh-7 cells exhibited the highest expression of AQP9, while the level in SMMC-7721 cells was the lowest among HCC cell lines. Therefore, Huh-7 and SMMC-7721 cells were used in further function study. Following the transfection with LV-AQP9, the expression level of AQP9 was significantly elevated in HCC cells (Figure 3B–3D).

### Overexpressed AQP9 inhibited proliferation, invasion, migration and EMT in HCC cells, but promoted cell apoptosis

**Figure 4:**
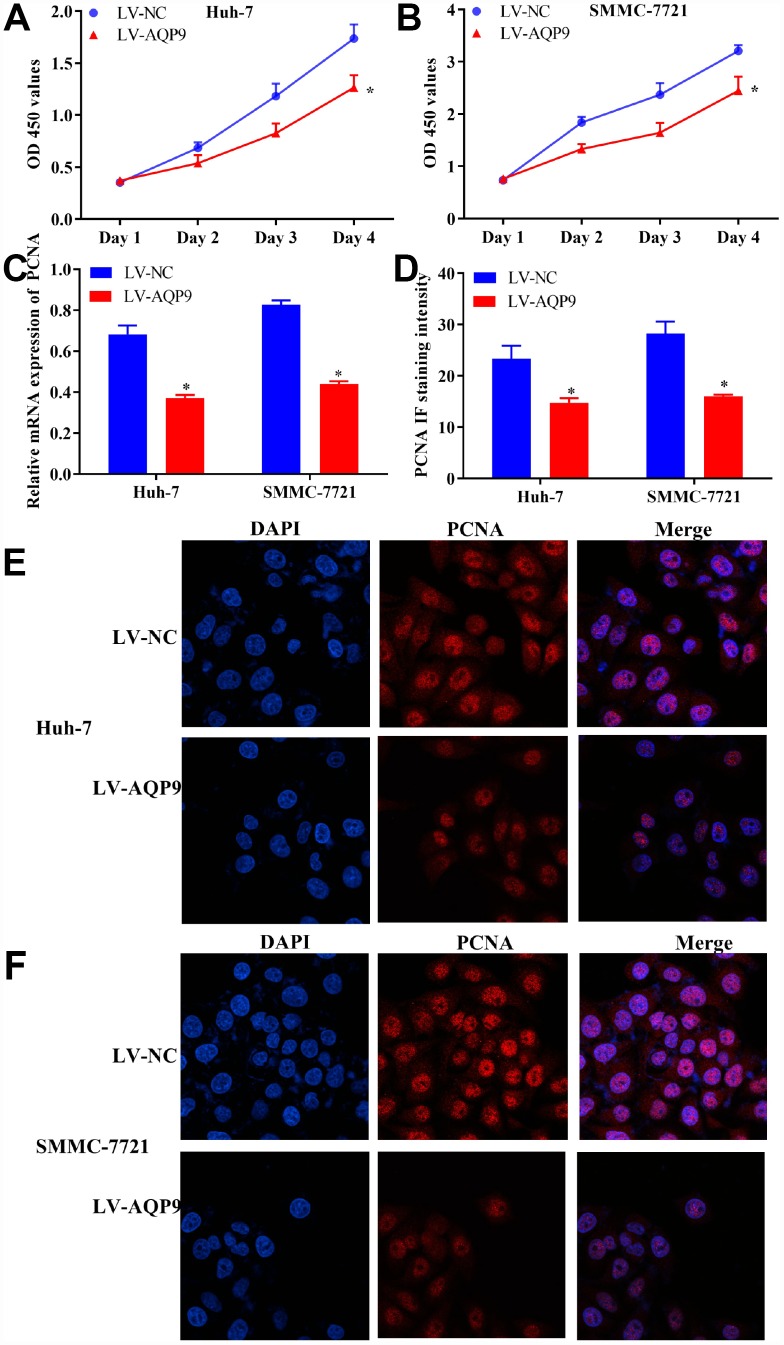
**Overexpression of AQP9 inhibited proliferation of HCC cells.** (**A** and **B**) The proliferative activity of Huh-7 and SMMC-7721 cells transfected with LV-AQP9 was determined by CCK-8 assay compared with the control. (**C**) The expression levels of PCNA in HCC cells were examined following the transfection with LV-NC or LV-AQP9. (**D**–**F**) The expression of PCNA in Huh-7 and SMMC-7721 cells transfected with LV-NC or LV-AQP9 were evaluated using immunocytochemistry analysis. The results were represented as mean ± SD. P<0.05 vs. LV-NC. Each experiment was repeated 3 times. AQP, aquaporin 9; HCC, hepatocellular carcinoma; NC, negative control.

To identify the regulatory functions of AQP9 on the biological behaviors of HCC cells, CCK8 assay was carried out. The data revealed that cell proliferation was remarkably suppressed in Huh-7 and SMMC-7721 cells transfected with LV-AQP9 (Figure 4A and 4B). In addition, the expression levels of proliferating cell nuclear antigen (PCNA) were examined. The results of RT-qPCR indicated that overexpression of AQP9 notably reduced the mRNA levels of PCNA in HCC cells (Figure 4C). Furthermore, immunocytochemistry analysis suggested that PCNA was predominantly expressed in the nucleus of HCC cells, and its expression levels were remarkably reduced following the transfection with LV-AQP9 (Figure 4D–4F). These findings revealed that overexpression of AQP9 was able to inhibit proliferation of HCC cells.

**Figure 5:**
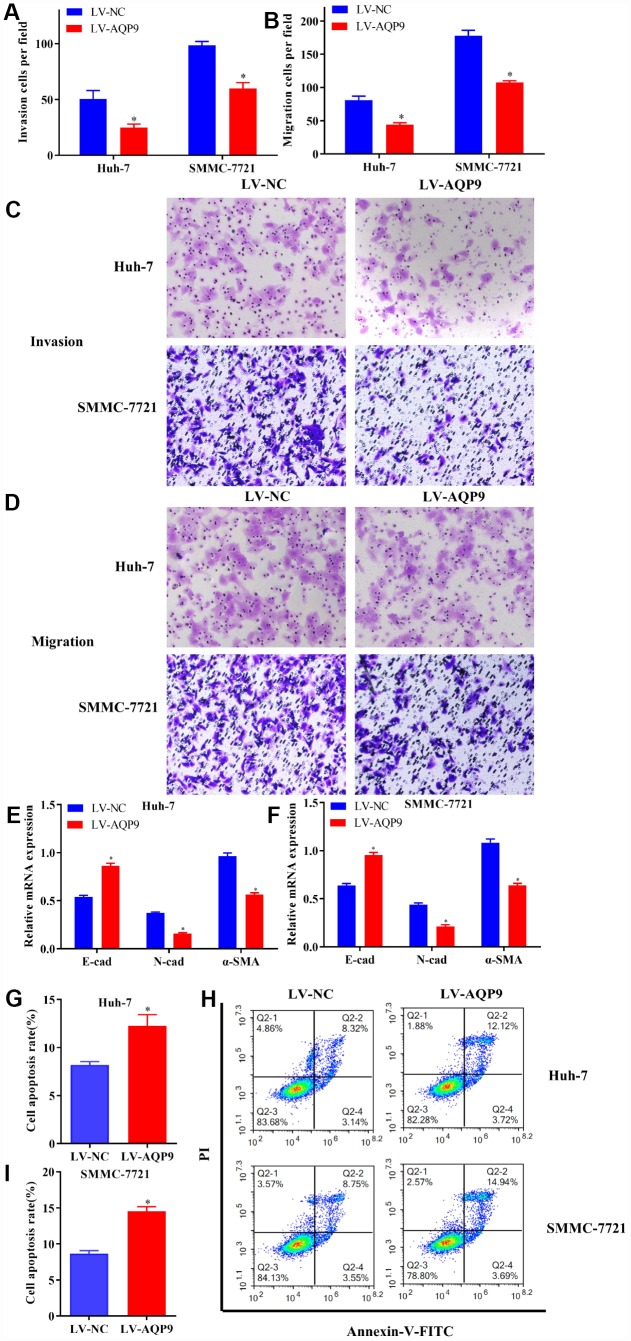
**Overexpressed AQP9 inhibited invasion, migration and EMT in HCC cells, but promoted cell apoptosis.** (**A** and **C**) The invasive abilities in Huh-7 and SMMC-7721 cells transfected with LV-NC or LV-AQP9 were determined by Transwell assay (magnificationx100). (**B** and **D**) Cell migration were examined following the transfection of LV-AQP9 (magnificationx100). (**E** and **F**) The mRNA levels of E-cad, N-cad and α-SMA in transfected Huh-7 and SMMC-7721 cells were evaluated using RT-qPCR. (**G**–**I**) The cell apoptosis rate in HCC cells transfected with LV-NC or LV-AQP9 was analyzed by flow cytometry. Data are shown as mean ± SD based on at least three independent experiments. The results were represented as mean ± SD. P<0.05 vs. LV-NC. Each experiment was repeated 3 times. AQP, aquaporin 9; HCC, hepatocellular carcinoma; NC, negative control; RT-qPCR, reverse transcriptase-quantitative polymerase chain reaction.

The progression of HCC was also assessed by examining cell invasion, migration and EMT. The results indicated that the invasion (Figure 5A and 5C) and migration (Figure 5B and 5D) of HCC cells transfected with LV-AQP9 were suppressed compared with the control. Furthermore, the expression levels of EMT-related markers, such as N-cad and α-SMA were significantly decreased, while E-cad was upregulated in HCC cells treated with LV-AQP9 (Figure 5E and 5F). Additionally, flow cytometry was performed to determine cell apoptosis following the transfection with LV-AQP9. Our data suggested that the apoptosis rate of Huh-7 and SMMC-7721 cells was remarkably increased after the treatment with LV-AQP9 (Figure 5G–5I). In summary, these findings indicated that overexpressed AQP9 could suppress cell proliferation, invasion, migration and EMT, while promoting cell apoptosis in HCC cells, suggesting AQP9 expression is associated with the biological behavior changes of HCC cells.

### Overexpression of AQP9 suppressed Wnt/β-catenin pathway in HCC cells

**Figure 6:**
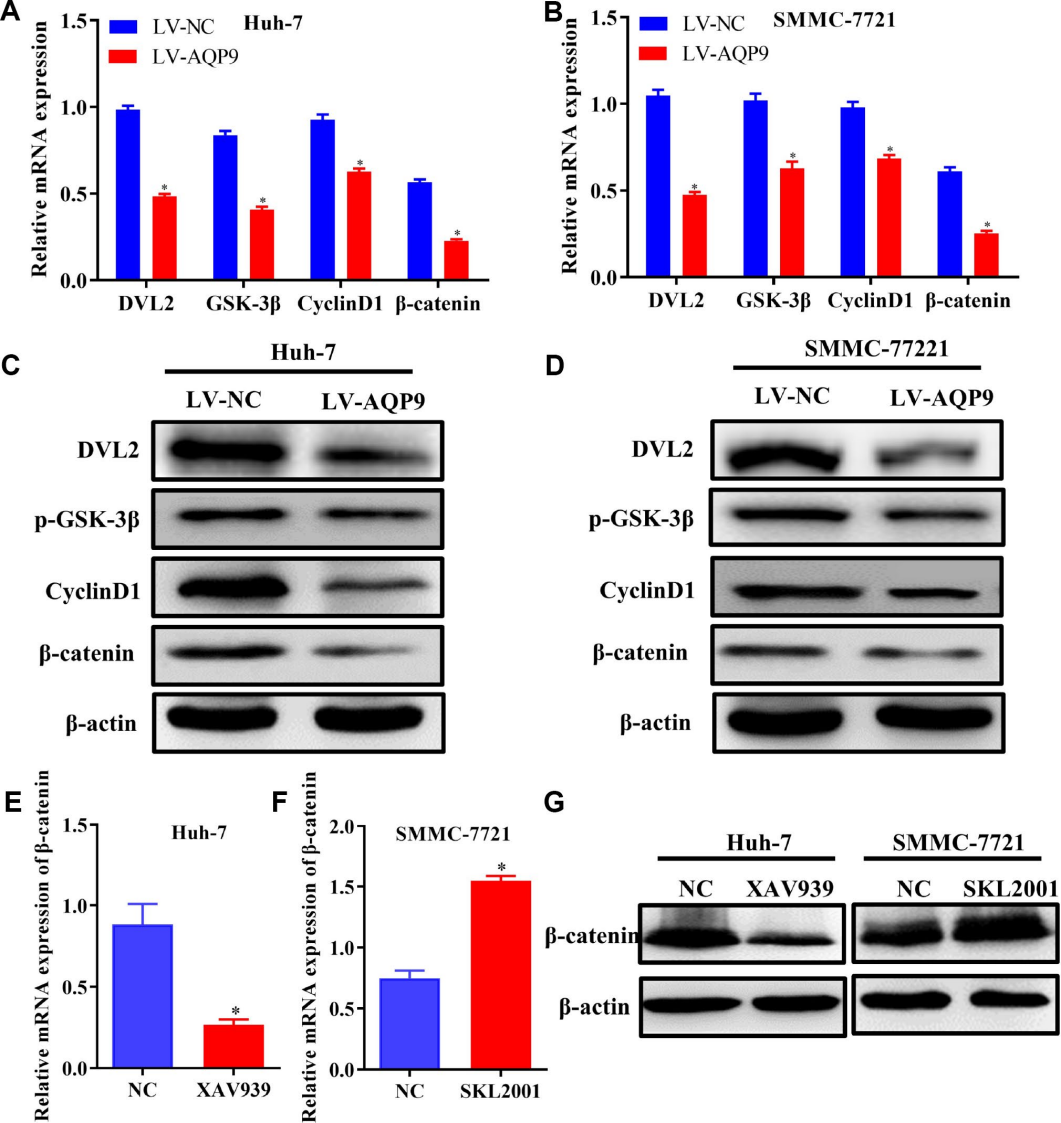
**Overexpression of AQP9 was able to suppress Wnt/β-catenin signaling.** (**A**–**D**) The mRNA and protein levels of DVL2, GSK-3β, CyclinD1 and β-catenin in Huh-7 and SMMC-7721 cells transfected with LV-NC or LV-AQP9 were examined by RT-qPCR and western blotting. (**E**) The expression of β-catenin in HCC cells treated with XAV939 were evaluated using RT-qPCR. (**F**) The mRNA levels of β-catenin in SMMC-7721 cells were determined following the treatment with SKL2001. (**G**) The protein levels of β-catenin in HCC cells were assessed by western blot analysis. The results were represented as mean ± SD. P<0.05 vs. NC or LV-NC. Each experiment was repeated 3 times. AQP, aquaporin 9; HCC, hepatocellular carcinoma; NC, negative control; RT-qPCR, reverse transcriptase-quantitative polymerase chain reaction.

Wnt/β-catenin signaling pathway plays essential roles during the development of HCC. The expression levels of related molecules involved in Wnt/β-catenin pathway were examined using RT-qPCR and western blotting. The results revealed that both mRNA and protein levels of DVL2, p-GSK-3β, CyclinD1 and β-catenin were significantly decreased in Huh-7 and SMMC-7721 cells transfected with LV-AQP9 (Figure 6A–6D). To further investigate the functions of Wnt/β-catenin signaling on the progression of HCC, Huh-7 and SMMC-7721 cells were treated with Wnt/β-catenin signaling inhibitor XAV939 or activator SKL2001, respectively. The data indicated that β-catenin was down-regulated in Huh-7 cells treated with XAV939 (Figure 6E and 6G) and up-regulated in SMMC-7721 cells following the treatment with SKL2001 (Figure 6F and 6G). These findings suggested that overexpressed AQP9 could exert its anti-tumor effects by suppressing Wnt/β-catenin signaling.

### Blockage of Wnt/β-catenin signaling inhibited the proliferation, invasion and migration of HCC cells, but promoted cell apoptosis

**Figure 7:**
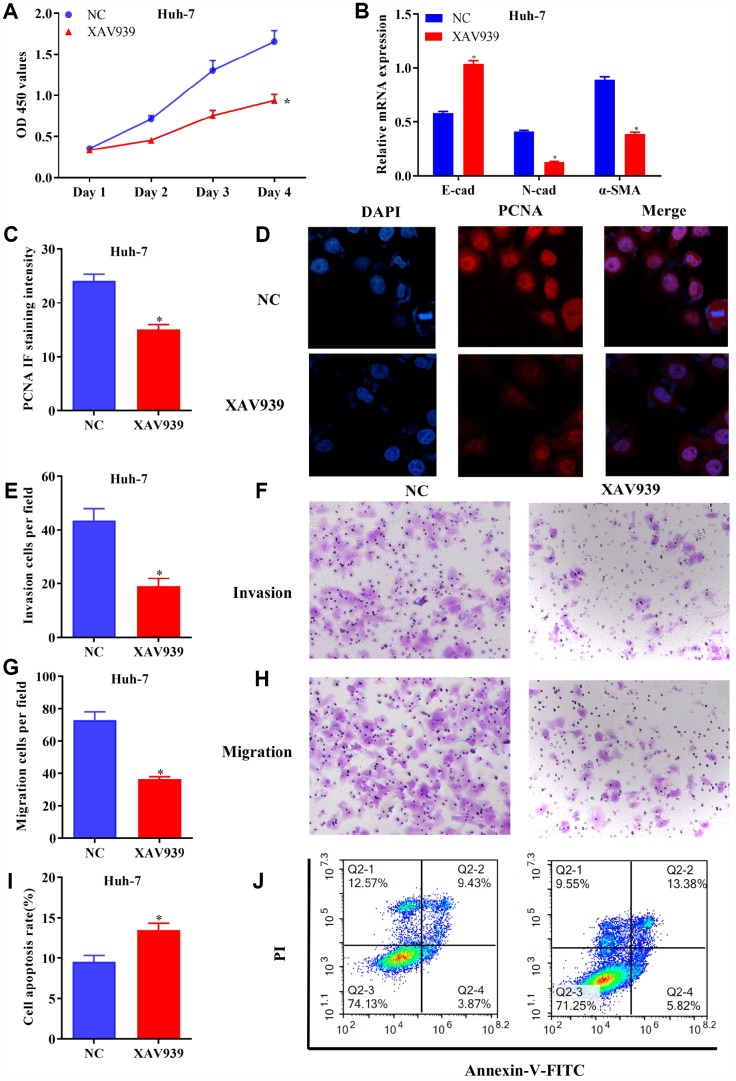
**Blockage of Wnt/β-catenin signaling inhibited growth and metastasis of HCC cells.** (**A**) The proliferation of Huh-7 cells treated with XAV939 was determined by CCK-8 assay. (**B**) The mRNA levels of E-cad, N-cad and α-SMA in transfected HCC cells were examined using RT-qPCR. (**C** and **D**) The expression of PCNA in Huh-7 cells were evaluated following the treatment with XAV939. (**E** to **H**) The invasive and migrative abilities of HCC cells treated with XAV939 were assessed by Transwell assay (magnificationx100). (**I** and **J**) Cell apoptosis following the treatment with XAV939 was determined using flow cytometry. The results were represented as mean ± SD. P<0.05 vs. NC. Each experiment was repeated 3 times. HCC, hepatocellular carcinoma; RT-qPCR, reverse transcriptase-quantitative polymerase chain reaction.

To further identify the regulatory roles of AQP9 in HCC via Wnt/β-catenin signaling, further function studies were conducted. The results revealed that cell proliferation was inhibited (Figure 7A), and EMT was suppressed (Figure 7B) in Huh-7 cells treated with XAV939. Furthermore, the expression levels of PCNA were downregulated in HCC cells following the treatment with XAV939 (Figure 7C and 7D). Additionally, cell invasion (Figure 7E and 7F) and migration (Figure 7G and 7H) were suppressed in Huh-7 cells treated with XAV939. Flow cytometry also indicated that the apoptosis rate of Huh-7 cells was remarkably decreased after the treatment with XAV939 (Figure 7I and 7J). Taken all together, as Wnt/β-catenin signaling plays key roles in the pathogenesis of liver cancer, AQP9 could affect the proliferation, invasion, migration and apoptosis of HCC cells through Wnt/β-catenin pathway.

### Activation of Wnt/β-catenin pathway reversed the effects of AQP9 on HCC

**Figure 8:**
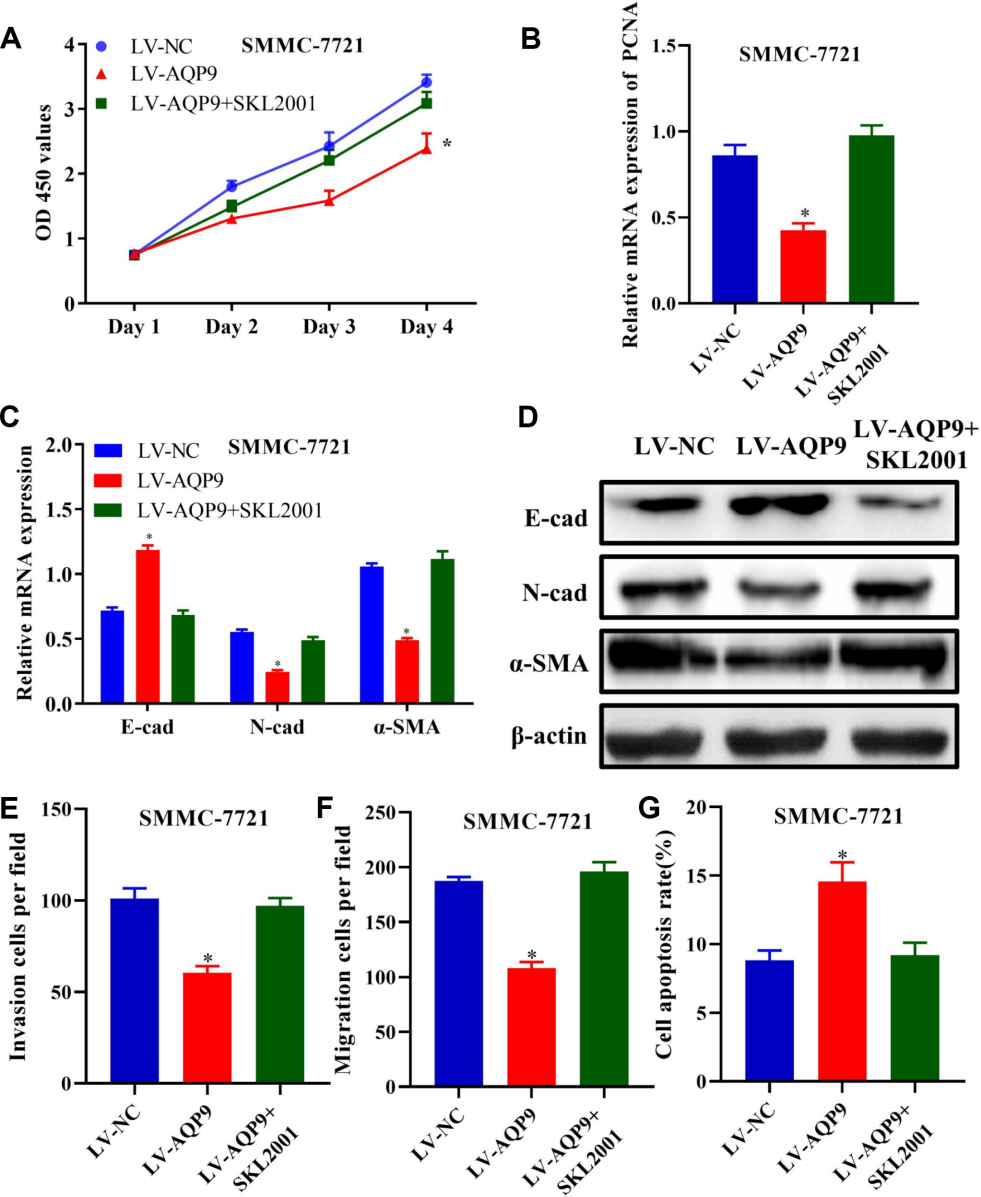
**Activation of Wnt/β-catenin pathway reversed the effects of AQP9 on HCC progression.** (**A**) The proliferation of SMMC-7721 cells transfected with LV-NC, LV-AQP9 or LV-AQP9+SKL2001 was determined. (**B**–**D**) The expression levels of PCNA, E-cad, N-cad and α-SMA in transfected HCC cells were examined using RT-qPCR. (**E** and **F**) The invasive and migration activities of SMMC-7721 cells were evaluated following the treatment with LV-NC, LV-AQP9 or LV-AQP9+SKL2001 (magnificationx100). (**G**) The apoptosis rate of transfected HCC cells was assessed by flow cytometry. The results were represented as mean ± SD. P<0.05 vs. LV-NC. Each experiment was repeated 3 times. AQP, aquaporin 9; HCC, hepatocellular carcinoma; NC, negative control; RT-qPCR, reverse transcriptase-quantitative polymerase chain reaction.

In order to confirm that overexpressed AQP9 could suppress the progression in HCC in a Wnt/β-catenin-dependent manner, SMMC-7721 cells were co-transfected with LV-AQP9 and SKL2001. The results revealed that cell proliferative activity (Figure 8A) and the expression of PCNA (Figure 8B) were significantly downregulated in HCC cells transfected with LV-AQP9, which was abolished by SKL2001 treatment. Similarly, EMT was inhibited in SMMC-7721 cells transfected with LV-AQP9, and these effects were reversed following co-transfection SKL2001 (Figure 8C and 8D). Furthermore, Transwell assays indicated that cell invasion and migration were suppressed by overexpressed AQP9, which were abrogated by the treatment with SKL2001. Flow cytometry also revealed that overexpression of AQP9 promoted cell apoptosis, and these effects were abolished by co-transfection with SKL2001. Our findings revealed that SKL2001 could reverse the effects caused by overexpressed AQP9 during the development of HCC, suggesting AQP9 may function as a tumor suppressor via suppressing Wnt/β-catenin signaling.

### Overexpression of AQP9 suppressed tumor growth *in vivo*

**Figure 9:**
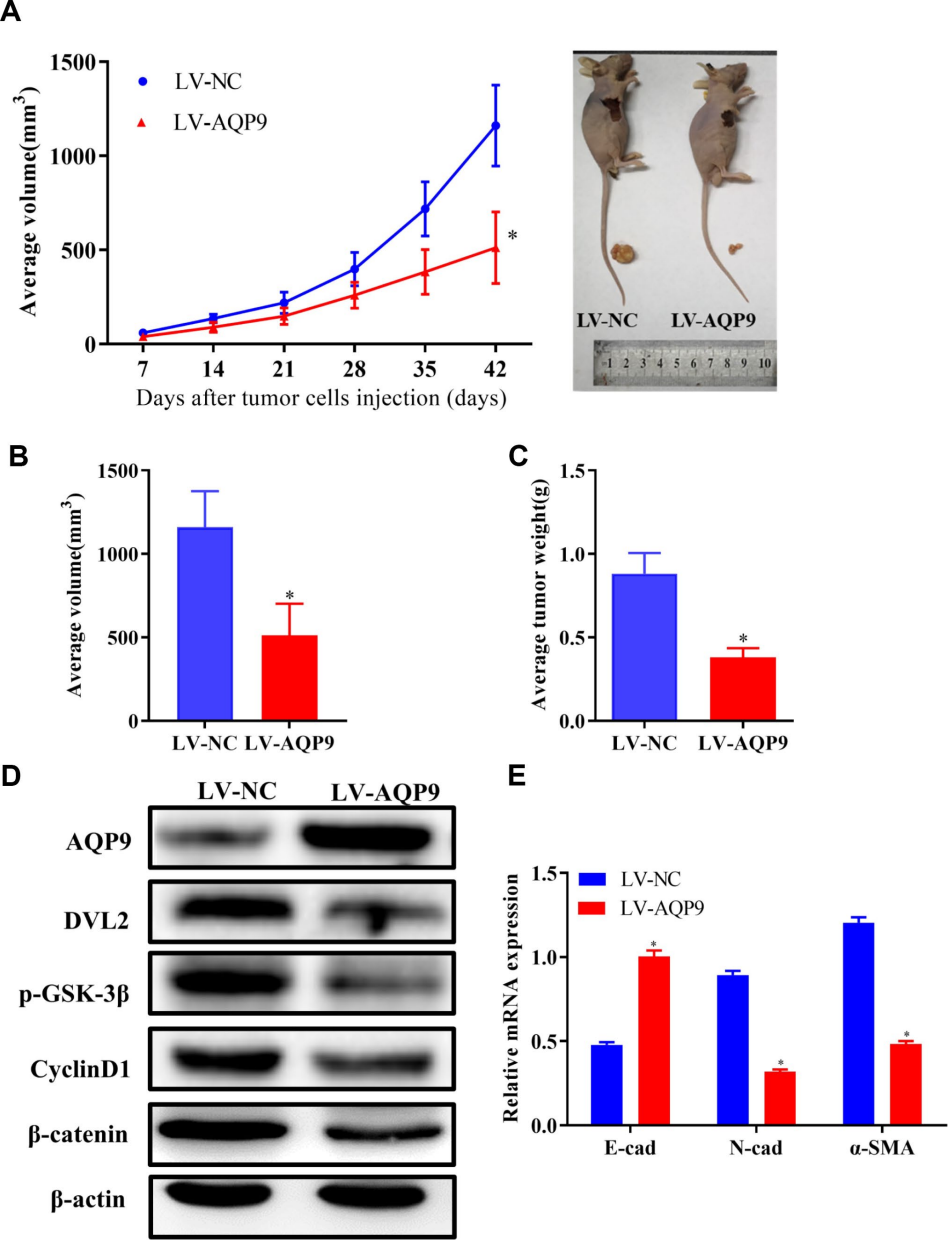
**Overexpression of AQP9 suppressed tumor growth of HCC *in vivo*.** (**A**) The growth curves of tumors from nude mice in LV-NC and LV-AQP9 group. (**B** and **C**). Orthotopic tumor volumes and weights at day 42 post-injection were calculated. (**D**) The protein levels of AQP9, DVL2, GSK-3β, CyclinD1 and β-catenin in isolated tumors were examined using western blotting. (**E**) The mRNA levels of E-cad, N-cad and α-SMA were determined by RT-qPCR. The results were represented as mean ± SD. P<0.05 vs. LV-NC. AQP, aquaporin 9; HCC, hepatocellular carcinoma; NC, negative control; RT-qPCR, reverse transcriptase-quantitative polymerase chain reaction.

To identify the novel functions of AQP9 *in vivo*, SMMC-7721 cells transfected with LV-NC or LV-AQP9 were injected into nude mice subcutaneously. Tumor volumes were measured weekly until day 42. Six weeks post-injection, the mice were sacrificed and the isolated tumors were examined. Mean value of tumor volume in LV-AQP9 group was significantly reduced compared with the control (Figure 9A and 9B). In addition, the tumor weight in LV-AQP9 group was notably decreased than LV-NC group (Figure 9C). Furthermore, the protein levels of AQP9, DVL2, GSK-3β, CyclinD1 and β-catenin in xenograft tumors were determined using western blot analysis. Our results revealed that the expression of AQP9 was significantly increased, while the levels of Wnt/β-catenin-associated molecules were remarkably reduced in LV-AQP9 group (Figure 9D). Moreover, EMT was inhibited in LV-AQP9 group compared with the control (Figure 9E). These findings suggested that AQP9 was a putative tumor suppressor during the development of HCC, which could affect the growth and metastasis of HCC cells by suppressing Wnt/β-catenin signaling. More importantly, AQP9/Wnt/β-catenin axis could be a potential therapeutic target during the treatment of HCC.

## DISCUSSION

AQP9 was predominantly located on the basolateral membrane of mammalian liver cells and serves essential roles on the absorption of arsenite, whose accumulation could result in damaged liver cells and HCC [8]. Our previous study has revealed that AQP9 is able to inhibit the development of HCC through activating PI3K/Akt and Caspase 3 signaling pathways [15]. However, the detailed functions of AQP9 on the pathogenesis of HCC have not been completely elucidated. In the present study, our results indicated that the mRNA levels of AQP9 were remarkably down-regulated in HCC patients with larger tumor diameter (≥5cm), lymph node metastasis and advanced TNM stage (III-IV). Furthermore, univariate analysis suggested that low-expression of AQP9 in HCC patients was associated with tumor size/number, TNM stage and lymph node/distant metastasis. In addition, log-rank test revealed that the five-year survival rate of AQP9 low-expression group was notably poorer compared with the high-expression counterpart, suggesting that AQP9 could be closely associated with the progression of HCC.

Recent studies have reported that impaired AQP9 expression may contribute to the development of numerous types of cancer, such as lung cancer and malignant brain tumour [16, 17]. For instance, the expression of AQP9 in prostate cancer was significantly increased compared with the adjacent tissues [16]. Furthermore, AQP9 could promote the proliferation, migration and invasion, whereas inhibit the apoptosis of prostate cancer cells. In addition, AQP9 serves essential roles during the progression of brain tumors. Studies have revealed that AQP9 was abundantly expressed in human glioma tissues, which is also correlated with the pathological grade of tumors [18]. AQP9 reduced the expression of E-cad and enhanced astrocytoma cell proliferation and migration through activating RAC serine/threonine protein kinase pathway [19]. Moreover, Huang et al. [20]. reported that colorectal cancer patients with high-expression of AQP9 exhibited better survival rate compared with the low-expression counterpart, suggesting that AQP9 could be used as an independent predictor of adjuvant chemotherapy for colorectal cancer. In our study, the proliferation of HCC cells was significantly down-regulated following the transfection with LV-AQP9. Furthermore, overexpression of AQP9 promoted the apoptosis of HCC cells. In consistence with these findings, our previous study also revealed that AQP9 suppressed the proliferation of SMMC-7721 cells by arresting them in S and G1 phase to induce apoptosis through up-regulating FOXO1 [21].

In malignant tumors, metastasis was detected in ≥90% of cancer patients [22]. HCC is one of the most common type of malignancies worldwide. The recurrence rate remains high and the prognosis is poor. It is mainly caused by highly proliferative and invasive ability of HCC cells [23, 24]. During metastasis, individual tumor cells disseminate from the primary tumor undergoes EMT and subsequently enter the blood circulation. EMT is characterized by the transformation of cell phenotype from epithelial to mesenchymal cells. Various epithelial and mesenchymal markers such as VI, N-cad and E-cad, Slug, Snail and Twist1 are associated with tumor progression [25, 26]. In our study, overexpression of AQP9 reduced the invasion and migration activity of HCC cells by down-regulating E-cad and upregulating N-cad and α-SMA, respectively. Therefore, overexpressed AQP9 could suppress the progression of HCC. Zhang et al. [27]. also revealed that FoxP4 promoted the migration and invasion of HCC cells. Jiang et al. [28]. indicated that PRTMT9 enhanced the invasion and metastasis of HCC via activating PI3K/Akt/GSK-3β/Snail signaling pathway.

Wnt/β-catenin signaling pathway is involved in cell differentiation, proliferation and apoptosis, such as regulation of intercellular junctions and maintenance of epithelial cell phenotype/tissue homeostasis [12]. Disruption within this pathway may lead to EMT. E-cad is required for the formation of intercellular junctions, and depletion of E-cad is the major cause of tumor invasion and metastasis [29]. Wnts are key regulators of cell proliferation, differentiation, adhesion and migration [30]. Impaired Wnt signaling may lead to the initiation and progression of cancer [31]. The classical Wnt/β-catenin pathway is characterized with the accumulation of cytoplasmic β-catenin, that interacts with TCF/LEF-Legless-PYGO DNA binding proteins to form a complex of transcriptional activators [32]. In our study, overexpressed AQP9 reduced the levels of DVL2, p-GSK-3β, CyclinD1 and β-catenin, suggesting that AQP9 could be a novel tumor suppressor by suppressing Wnt/β-catenin signaling pathway. In further studies, inhibition of Wnt/β-catenin signaling down-regulated cell proliferation, invasion and migration, while promoted cell apoptosis of HCC cells. These findings suggested that blockage of Wnt/β-catenin pathway could suppress the development of HCC.

Furthermore, Liu et al. [33]. reported that SOX11 inhibited Wnt/β-catenin signaling pathway to modulate the apoptosis and cell cycle of HCC cells. Studies have also revealed that overexpression of microRNA-194 lead to downregulation of β-catenin, Wnt3a, N-cad and up-regulation of E-cad in HCC cells. Moreover, elevation of miR-194 inhibited EMT, cell proliferation, invasion and migration via suppressing Wnt/β-catenin signaling [34]. Lei et al. [35]. also found that silencing of FOXD2-AS1 activates Wnt/β-catenin signaling, affecting cell proliferation, apoptosis, migration and EMT and consequently promoting the progression of HCC. In this study, in order to further confirm that overexpressed AQP9 may inhibit the progression in HCC by suppressing Wnt/β-catenin signaling, SKL2001 was used to activate Wnt/β-catenin pathway in HCC cells following the transfection with LV-AQP9. The results revealed that activation of Wnt/β-catenin signaling reversed the effects caused by overexpressed AQP9 on the biological behaviors of HCC cells.

In vivo, the ectopic tumor volume and weight were significantly reduced in LV-AQP9 group compared with the control, and the levels of DVL2, GSK-3β, CyclinD1 and β-catenin were down-regulated in tumor tissues. Moreover, the expression of EMT-related markers was decreased in LV-AQP9 group. These findings indicated that overexpressed AQP9 could inhibit tumor growth in vivo through suppressing Wnt/β-catenin signaling and down-regulating the expression of EMT-related molecules.

In summary, our data revealed that AQP9 was down-regulated in HCC tissues and cells, which was also associated with the clinical features and prognosis of patients with HCC. Overexpressed AQP9 was able to inhibit the growth and metastasis of HCC cells by suppressing Wnt/β-catenin pathway. More importantly, AQP9 could be a novel therapeutic target for the treatment of HCC patients.

## MATERIALS AND METHODS

### Patient samples

A total of 50 paired HCC specimens (42 male and 8 female; average age, 53.26±10.22 years) were obtained from the patients who underwent surgical resection at the Second Affiliated Hospital of Chongqing Medical University (Chongqing, China). None of the patients received immuno-, chemo- or radio-therapy prior to the operation. The clinicopathological characteristics of HCC patients were reviewed by two independent pathologists. All the samples were snap-frozen using liquid nitrogen until further use. The patients were divided into two groups according to the mean expression level of AQP9 (AQP9 high/low expression group). Informed written consents were signed by all the patients. The protocol of this study was approved by the Ethics Committee of the Second Affiliated Hospital of Chongqing Medical University.

### Cell culture and transfection

Normal human hepatocyte (LO2) as well as HCC cells (HLE, Huh-7, HepG2 and SMMC-7721) were purchased from the Cell Culture Center, Chinese Academy of Medical Sciences (Shanghai, China). The cells were cultured using Dulbecco’s modified Eagle’s medium (DMEM; Gibco, USA) supplemented with 10% fetal bovine serum (FBS; Gibco, USA) and incubated at 37°C in a humidified atmosphere with 5% CO_2_. Huh-7 and SMMC7721 cells were transfected with AQP9 overexpressing vector (LV-AQP9) or the control (LV-NC). Subsequently, the cells were selected with 1 μg/mL puromycin for four weeks. The cells were harvested 48 h post-transfection for use in further experiments. To inhibit or induce Wnt/β-catenin pathway, Huh-7 cells were treated with 10 mM XAV939 (MedChemExpress LLC, Monmouth Junction, NJ, USA), and SMMC-7721 were treated with 40 μM SKL2001 (MedChemExpress LLC, Monmouth Junction, NJ, USA), respectively.

### Reverse transcription-quantitative polymerase chain reaction (RT-qPCR)

Total RNA was extracted from tissues or cells using TRIzol^®^ reagent (Invitrogen; Thermo Fisher Scientific, Inc., Carlsbad, CA, USA) according to the manufacturer's protocols. Extracted RNA was reverse transcribed by PrimeScript RT kit with gDNA Eraser (Takara Biomedical Technology Co., Ltd., Beijing, China) according to the manufacturer's protocols. Quantitative PCR was performed using SYBR Green PCR Master Mix (Takara Biomedical Technology Co., Ltd., Beijing, China) according to the manufacturer’s protocols. Relative expression was normalized to endogenous Glyceraldehyde 3-phosphate dehydrogenase (GAPDH). The forward and reverse primer sequences were as follows: AQP9 forward, 5′-AAATAAACCTCCTTGGCCTGA-3′ and reverse, 5′-GCAACAAACATCACCACACC-3′; E-Cad forward, 5′-CAATGGTGTCCATGGAACA-3′ and reverse, 5′-CCTCCTACCCTCCTGTTCG-3′; α-SMA forward, 5′-TCCCTTGAGAAGAGTTACGAGTTG-3′ and reverse, 5′-ATGATGCTGTTGTAGGTGGTTTC-3′; N-Cad forward, 5′-CAGTATCCGGTCCGATCTGC-3′ and reverse, 5′-GTCCTGCTCACCACCACTAC-3′; DVL2 forward, 5′-AGTCAGCTCTCATGTTGAGGGT-3′ and reverse, 5′-TACCCAGCCCACACCTTCTT-3′; GSK-3β forward, 5′-CCGACTAACACCACTGGAAGCT-3′ and reverse, 5′-AGGATGGTAGCCAGAGGTGGAT-3′; CyclinD1 forward, 5′- GAGACCATCCCCCTGACGGC-3′ and reverse, 5′-TCTTCCTCCTCCTCGGCGGC-3′; β-catenin forward, 5′-TGCAGTTCGCCTTCACTATG-3′ and reverse, 5′-ACTAGTCGTGGAATGGCACC-3′; and GAPDH forward, 5′-GCAAGAGCACAAGAGGAAGA-3′ and reverse, 5′-ACTGTGAGGAGGGGAG ATTC-3′. Relative expression levels were calculated using 2^-ΔΔCq^ method.

### Western blotting

Total protein was extracted from tissues or cells using RIPA buffer (Beyotime Biotechnology, Shanghai, China). Protein concentrations were determined using a BCA Kit (Beyotime Biotechnology). Equal amounts (40 μg) of proteins were separated by 12% sodium dodecyl sulfate-polyacrylamide gel electrophoresis and transferred onto polyvinylidene fluoride membranes (Millipore, Billerica, MA, USA). Subsequently, the membranes were blocked with 5% (w/v) non-fat milk in TBST buffer (Beyotime Biotechnology) at 37°C for 1 h. The membranes were then incubated with anti-AQP9 (1:1000; cat. no. ab15129), anti-E-Cad (1:5000; cat. no. ab40772), anti-α-SMA (1:2000; cat. no. ab124964), anti-N-cad (1:5000 dilution; cat. no. ab18203), anti-β-catenin (1:4000; cat. no. ab6302), anti-CyclinD1 (1:100; cat. no. ab16663), anti-p-GSK-3β (1:10000; cat. no. ab75814), anti-DVL2 (1:1000; cat. no. ab22616), anti-β-actin (1:5000; cat. no. ab179467) antibodies (Abcam, Cambridge, MA, USA) at 4°C overnight. Following three washes in TBST for 10 min, the membranes were incubated with horseradish peroxidase-conjugated goat anti-mouse IgG (1:1,000; cat. no. 7076) or goat anti-rabbit IgG (1:1,000; cat. no. 7074) antibodies (Cell Signaling Technology, Danvers, MA, USA) at 37°C for 1 h. The protein bands were visualized by an enhanced chemiluminescence kit and quantified by densitometric analysis using ImageJ software (NIH, Bethesda, MD, USA).

### Immunohistochemistry (IHC) staining

Patient samples were fixed and embedded in paraffin. The sections were deparaffinized and hydrated, then blocked with endogenous peroxidase and pretreated with citrate buffer for antigen retrieval. The sections were rinsed with PBS and then incubated with anti-AQP9 (1:500; cat. no. ab15129; Abcam, Cambridge, MA, USA) at 4°C overnight, followed by incubation with secondary antibody at room temperature for 30 min. After three washes with PBS, staining intensity was measured by selecting five random fields per section and analysed using Image-Pro Plus software (Media Cybernetics, Rockville, MD, USA).

### Immunofluorescence analysis

Transfected Huh-7 and SMMC-7721 cells were seeded onto a 6-well culture plate at the density of 2x10^5^ cells/well. After the incubation for 12 h, the culture medium was replenished. The cells were rinsed with PBS for five min and fixed with 4% paraformaldehyde solution for 15 min. Then the cells were treated with 2.5% Triton for 10 minutes to permeabilize the membrane and blocked with goat serum at 37°C for 1 h. Subsequently, the cells were incubated with anti-PCNA (1:100; cat. no. ab92552; Abcam, Cambridge, MA, USA) antibody at 4°C overnight, followed by the incubation with secondary antibody at room temperature for five min. DAPI nuclear staining solution (1 μg/mL) was added at room temperature for five min. The cells were immersed with fluorescent quencher and subjected to fluorescence microscope.

### Cell proliferation assay

Transfected Huh-7 and SMMC-7721 cells were seeded at a density of 5x10^3^ cells/well and were examined on day 1, 2, 3 and 4 post-transfection according to the manufacturer’s protocols. Cell viability was evaluated using Cell Counting kit-8 assay (CCK-8; Beyotime, Shanghai, China). Briefly, CCK8 solution was added to each well and the cells were incubated for an additional 2 h. The absorbance at 450 nm was measured using a microplate reader (9200, Bio-Rad Laboratories, Hercules, CA, USA) according to the manufacturer's protocols.

### Transwell migration and invasion assay

Two days post-transfection, Huh-7 and SMMC-7721 cells were harvested. The migration and invasion of cells were examined using a Transwell assay. For cell migration assay, a total of 5x104 cells in serum free medium were placed onto the upper chamber (pore size=8μm; BD Biosciences, Franklin Lakes, New Jersey, USA). For cell invasion assay, 1x105 cells were added into the upper chamber which was pre-coated with Matrigel^®^ (Sigma-Aldrich, St. Louis, MO, USA). Then, 500 μl of culture medium containing 10% FBS was added into the lower chamber. Following overnight incubation, non-migratory/-invasive cells were removed using a cotton swab. The migrated/invaded cells in the lower chamber were fixed using 4% paraformaldehyde and stained with 0.5% crystal violet. The numbers of cells were counted within five randomly selected fields by an inverted light microscope (magnificationx100, Olympus Corporation, Tokyo, Japan).

### Apoptosis analysis

Transfected Huh-7 and SMMC-7721 cells were placed onto a 6-well culture plate at the density of 2x10^5^ cells/well. After 12 h, the medium was replenished. The cells were centrifugated and resuspended with pre-chilled 75% ethanol prior to overnight fixation at -20°C. Subsequently, the cells were resuspended using 450μL PBS supplemented with 50μL propidium iodide (PI, 0.5 mg/mL; Beyotime Biotechnology) and incubated at 37°C for 30 min. Then, 5 μL Annexin-V-fluorescein isothiocyanate (FITC; Beyotime Biotechnology) was used to stain the cells. The apoptosis was analyzed using a flow cytometer (BD Biosciences, USA).

### Animal study

A total of eight female BALB/C nude mice (4-6 weeks old) with a weight of 17-22 g were obtained from the Experimental Animal Center of Chongqing Medical University (Chongqing, China). The protocols were reviewed and approved by the Research Ethics Committee of Chongqing Medical University (Chongqing, China). The mice were routinely housed under a temperature- (22±2°C) and humidity-controlled (80%) atmosphere, with a 12-h dark/light cycle and libitum access to food/water for at least three days before the operation. SMMC-7721/LV-AQP9 or SMMC-7721/LV-NC cells (2 x 10^6^) were suspended in 200 μl PBS and injected subcutaneously into right side of the armpit regions of mice. Six weeks post-injection, the mice were sacrificed by decapitation, and the tumors were isolated and examined. Tumor volume was calculated using the following formula: V (mm^3^) = 0.5 x length x width^2^. The tumor tissues were snap-frozen in liquid nitrogen until further use.

### Statistical analysis

Data were presented as the means ± standard deviation and were analyzed using Graphpad Prism v7.0 software (Graphpad Software Inc., La Jolla, CA, USA). A t-test (two-sided) or one-way analysis of variance (ANOVA) was used for statistical analysis. A student-Newman-Keuls test was performed as a post-hoc test following ANOVA. Count data were processed by chi-square test. Overall survival was analyzed using Kaplan-Meier survival test. All the experiments were performed at least three times. P<0.05 was considered to indicate a statistically significant difference.
